# A comparative study of silver electrodeposition from pyrophosphate-cyanide and high concentration cyanide electrolytes in the presence of brighteners

**DOI:** 10.3906/kim-1907-80

**Published:** 2020-04-01

**Authors:** Hatice Kübra AKBEN, Servet İbrahim TİMUR

**Affiliations:** 1 Department of Metallurgical and Materials Engineering, İstanbul Technical University, İstanbul Turkey; 2 Department of Metallurgical and Materials Engineering, Engineering Faculty, İstanbul Gedik University, İstanbul Turkey

**Keywords:** Electrodeposition, silver, cyanide, brighteners, potassium antimony tartrate, 2-mercaptobenzothiazole

## Abstract

A study of the electrodeposition of silver from 2 different types of electrolytes; (1) neutral pyrophosphatecyanide electrolyte and (2) alkaline high concentrated cyanide electrolyte in the presence of a variety of additives such as 2-mercaptobenzothiazole, potassium selenocyanate, and potassium antimony tartrate was performed. Influence of additives and cyanide concentration on microstructure and kinetics of the cathodic processes were studied. A brightener couple, 2-mercaptobenzothiazole and potassium antimony tartrate, were combined within this investigation and detected to be highly effective for silver electrodeposition. The rapid increase in current density at the same potential interval related to grain refinement effect of potassium antimony tartrate was shown. The cyclic organic compound, 2-mercaptobenzothiazole, polarizes the reduction to high cathodic potential in pyrophosphate electrolyte. However, the sufficient levelling effect required for the mirror-bright appearance seems to be related to the high polarizing effect of the high concentration cyanide content. In the case of pyrophosphate electrolytes, sufficient levelling cannot be achieved, so semigloss coatings are obtained. The low cathodic potential electrodeposition of silver in pyrophosphate electrolyte, which is found to proceed by 3D instantaneous nucleation, is polarized to high cathodic potentials and grows into 3D progressive nucleation and diffusion-controlled growth in high concentration cyanide electrolyte.

## 1. Introduction

Electrodeposition of silver is a common industrial process in the manufacture of mirrors, in decorative applications, electronic applications, bearings, hot gas seals, and many other applications because of the high reflectivity of the surface and the highest electrical and thermal conductivity of all metals. The significant technological concern is to achieve a compact, adherent, and smooth silver electrodeposition. Today, industrially, this type of silver coating is still achieved by electrodeposition in baths if highly concentrated cyanide, though this process poses the problems of high toxicity of cyanide and high cost of wastewater treatment. To overcome these problems, researches on less toxic compounds for silver electroplating have been receiving great attention. Electroplating baths of nitrate [1–4], uracil [5], thiourea [6], 2-hydroxypyridine [7,8], 5,5-dimethylhydantoin [9,10], ferrocyanide-thiocyanide [11,12], ionic liquids [13–15] have been proposed. However, industrial use of these baths is still restricted because their use does not achieve coating of high brightness, compactness, and adhesion; the baths lack stability and the complexing agents are expensive. The other type of electrolyte, pyrophosphate-cyanide, contains pyrophosphate buffer salt and metal-cyanide complex ion as a source of metal without free cyanide. It is also known as low cyanide concentration bath [16–19]. This type of bath has been researched especially in patent literature [20–22]. Since high cyanide concentration baths cause the decomposition of photoresists, the baths without free cyanide are used in the manufacture of printed circuit boards [23]. Another reason for this is, the semigloss finish of the coatings produced by this chemical is usually preferred in semiconductor packages for image recognition [24].

Nowadays, also silver alloy electrodeposition, especially Cu-Ag [25–27] and Sn-Ag [28–30] coatings, has become a widely studied research area for various industrial purposes, mainly due to combining of mechanical strengthening with high electrical conductivity and corrosion resistance by a low cost, versatile technique. The electrodeposition technique is well designed for obtaining smooth and coherent deposits by means of using organic and inorganic additives to improve electrocrystallization and growth stages. The most well-known mechanism for these brighteners, added in low ratios, is the inhibition of the electrodeposition by way of adsorption on the protrusions of the cathode surface which are the rapid growth points [31,32]. Therefore, growth proceeds in the recessed areas. The other most known role is grain refinement effect which also improves the specular reflection ratio of the surface and leads to an increase in brightness [33]. The widely used brighteners; potassium antimony tartrate (PAT), 2-mercaptobenzothiazole (MBT), potassium selenocyanate (KSeCN) were researched in this study. The effect of PAT on the brightness of silver is related to the increase in the number of nuclei and not to the prevention of vertical growth [11,34]. Also, strong adsorption ability of PAT on the silver surface was demonstrated but proved less stable than that of an organic brightener [35]. High brightness effect of 2-mercaptobenzothiazole’s (MBT) was only observed for electroless nickel plating [36]. It was indicated that the brightener effect of selenium is related to its catalytic effect at the electrode/electrolyte interface which improves the silver deposition rate [37]. This behaviour was observed as a rising current-time transient. However, the combined effect of these additives on silver electrodeposition has not been considered yet. A systematic investigation of the combined effect of these additives (PAT, MBT, KSeCN) as compared to the cyanide concentration for silver electrodeposition is lacking in the literature.

In this paper, silver electrodeposition with inorganic brighteners KSeCN, PAT, and organic brightener; MBT were investigated along with pyrophosphate-cyanide (pyro) and traditional high concentration cyanide electrolyte (h-cya). After determining the most efficient brightener couple, the synergistic effects of brighteners on the reduction and nucleation mechanisms were investigated by cyclic voltammetry and chronoamperometry, respectively. The influences of cyanide concentration on plating quality were also presented.

## 2. Materials and methods

### 2.1. Plating methods and materials

Distilled water and analytical grade chemicals were used to prepare all the electrolytes.

KSeCN, MBT, and PAT were acquired from Sigma Aldrich.

Hull cell experiments were performed to determine the c.d. (current density) ranges within which matt, semibright, or bright silver coatings are formed. Standard Hull cell in a 267 mL capacity was utilized to determine the operating c.d. range at a constant current of 2 A at 25 °C for 2 min unless otherwise stated. A magnetic stirrer at a speed of 350 rpm was used during all galvanostatic plating and Hull cell experiments. The stirring speed was kept constant in all experiments. Therefore, the agitation effect will not be discussed in this paper. The galvanostatic plating cell had a volume of 250 cm^3^ . The bath compositions and operating conditions used in Hull cell experiments are presented in Table 1.

**Table 1:** The bath compositions used in Hull cell experiments.

Electrolyte composition	Pyro electrolyte (g L^-1^)	H-Cya electrolyte (g L^-1^)
KAg(CN)_2_	80	80
KCN	0.5	60
Potassium pyrophosphate	80	-
Sodium tetraborate decahydrate	30	-
KOH	-	7.5
KSeCN	0.5 ppm	0.5 ppm
PAT	0.5	0.5
MBT	0.05	0.05

The Hull cell and galvanostatic plating experiments were carried out using a 2-electrode configurations consisting of the platinized titanium mesh electrode acting as the anode and silver-plated copper electrode acting as the cathode in the electrolyte. 2 ×4 cm^2^ cathodes were used in galvanostatic plating. Copper cathodes were firstly activated by immersion in 1M H_2_SO_4_ for 10 s and then rinsed with distilled water. After adequate rinsing, silver strike bath (4 g L^-1^ KAgCN_2_ , 80 g L^-1^ potassium pyrophosphate, 0.5 ppm KSeCN, 0.5 A dm^-2^ , 25 °C) was utilized for electrodeposition of strike coating to prevent immersion deposition.

### 2.2. Electroanalytical characterization

The platinum working electrode having a 0.09 (0.3 ×0.3) cm2 surface area and spiral platinum counter electrode were used in a standard 3-electrode cell (50 cm^3^) configuration. All potential values were reported versus the standard calomel electrode. The cyclic voltammetry experiments were carried out at a sweep rate of 50 mV s^-1^ . The third cycle corresponding to a steady voltammogram was represented in all voltammograms. For chronoamperometric analysis, first, the potential was held at 0.0 V for 10 s then stepped up to working potential to initiate nucleation and growth on the electrode. All the electrochemical analyses were conducted by Gamry PCI4/750TM potentiostat.

### 2.3. Characterization

The morphology of the electrodeposits coated with or without additives and free cyanide was analysed by field emission scanning electron microscope (FEG-SEM, Jeol JSM 7000F).

A reflectance spectrophotometer (Scinco S3100-SA13) was applied for the reflection analysis of the deposits. The reflected beam was collected using an integrated sphere. This type of reflectance spectrophotometer gives the dependencies of total reflection and diffuses reflection as a function of the wavelength of the visible light.

## 3. Results and discussion

### 3.1. Hull cell testing

**Figure 1:**
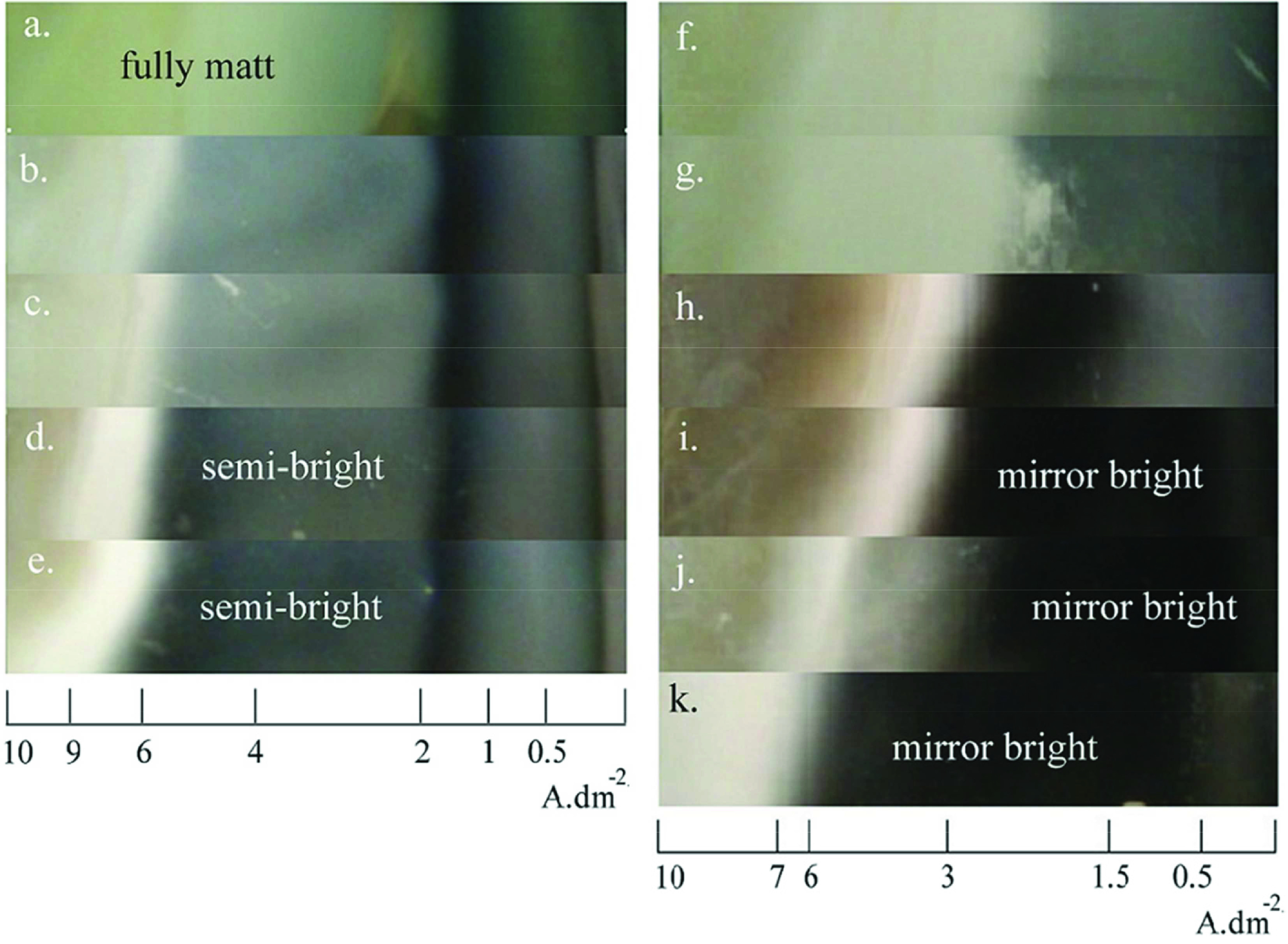
Hull cathode images of electrodeposits from different electrolytes: pyro electrolyte with a) no additive and borax, b) KSeCN, c) MBT+KSeCN, d) MBT, e) PAT+ MBT, and h-cya electrolyte with f) no additive, g) KSeCN, h) MBT+KSeCN, i) MBT, j) PAT, k) PAT+MBT.

The effects of electrolyte content and the addition of KSeCN, MBT, PAT additives on the bright c.d. range is presented with the digital images of Hull cathodes and Hull cell ruler. The images of pyro baths are given in Figure 1. All electrolytes result in matt and semibright coatings. As shown in Figure 1a almost fully burnt and smoky deposit occurs without brightener addition. KSeCN included electrolytes (Figure 1b and 1c) form more smoky coating according to the MBT and/or PAT-containing electrolytes (Figure 1d and 1e). However, the less hazy semibright result is achieved with the MBT+PAT brightener couple (Figure 1e). It is observed that the improvement in brightness is up to 6.0 A dm^-2^ c.d. with the addition of brighteners.

The effect of the same brighteners (MBT, PAT, KSeCN) on high concentration cyanide electrolyte (hcya) was investigated. Figure 1f demonstrates smoky and matt coating without brighteners. The electrolytes containing KSeCN, as shown in Figures 1g and 1h, form a more hazy coating in lower c.d. ranges. Only MBT or PAT addition (Figure 1i and 1j) gives mirror-bright coating in lower c.d. range (0.5–3.0 A dm^-2^) whereas the electrolyte containing MBT+PAT brightening couple electroplates a mirror-bright deposit in widest c.d. range, 0.5–6.0 A dm^-2^ as seen in Figure 1k.

### 3.2. Deposit morphology

**Figure 2:**
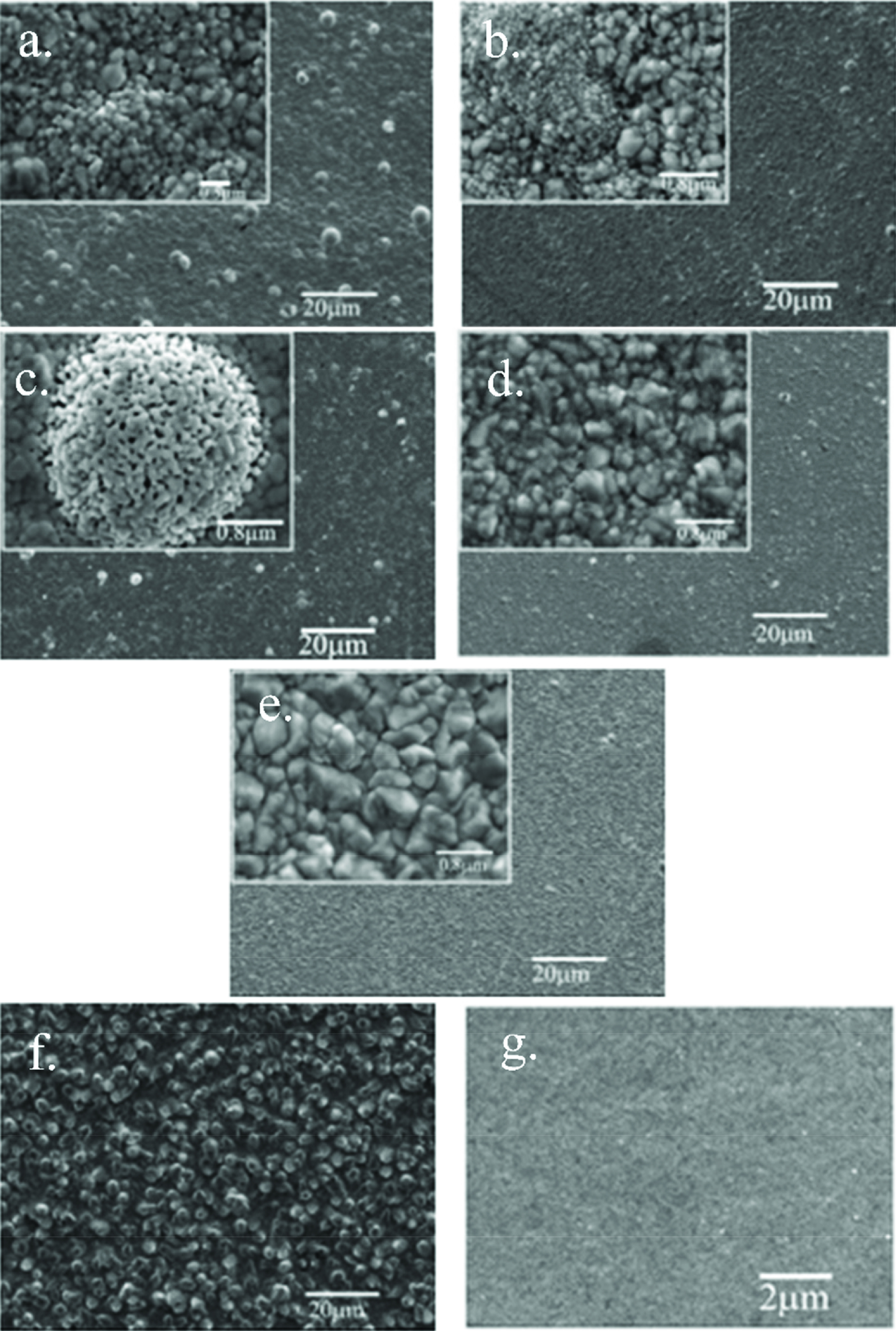
FESEM images of electrodeposits from a) pyro electrolyte (KAg(CN)_2_+0.5 g L^-1^ KCN+potassium pyrophosphate+borax), b) pyro electrolyte with MBT, c) pyro electrolyte with PAT, d) pyro electrolyte with MBT+PAT at 2 A dm^-2^ c.d., e) pyro electrolyte with MBT+PAT at 6 A dm^-2^ c.d., f) h-cya electrolyte without brighteners, g) h-cya electrolyte with MBT+PAT.

Figure 2 shows different effects of the additives on the microstructure via FESEM analysis of the samples coated by galvanostatic electrodeposition. Pyro electrolyte leads to high globular growth at a c.d. of 2 A dm^-2^ , as seen in Figure 2a. This nodular growth decreases with the addition of MBT to the electrolyte (Figure 2b). However, the structure has irregular grain size and the coating is still visually semibright. PAT has a strong grain refinement effect as seen in Figure 2c. Adding MBT+PAT brightener couple together (Figure 2d) gives a more homogenous grain refinement effect than with only MBT addition and lesser nodular growth than only PAT addition. However, the additives do not provide the desired smooth and fine microstructure. When the c.d. increases to 6.0 A dm^-2^ , the nodular growth decreases but leads to the growth of the grains as seen in Figure 2e. In that case, the semi-gloss coating is cloudier.

H-cya electrolyte without the addition of any brightener leads to rod-type elongated growth, as seen in Figure 2f. The addition of the MBT+PAT brightener couple to this electrolyte provides a smooth morphology of nanometric small grains (Figure 2g) which is the microstructure of a mirror-bright silver deposit.

### 3.3. Cyclic voltammetry

The brighteners’ effects on the kinetics of electrochemical deposition were investigated by cyclic voltammetry analysis. Table 2 presents the solutions and their abbreviations used in the electrochemical analysis.

**Table 2:** Electrolyte compositions used in electrochemical analyses.

Electrolyte (g L^-1^)	KAg(CN)_2_	K4P2O7 (Pyrophosphate)	KCN	PAT	MBT	Borax
S1	8	-	-	-	-	-
S2	8	80	-	-	-	-
S3	8	80	0.5	-	-	-
S4	8	80	0.5	-	-	30
S5	8	80	0.5	0.5	-	30
S6	8	80	0.5	-	0.05	30
S7	8	80	0.5	0.5	0.05	30
S8	8	80	5	0.5	0.05	30
S9	8	80	0.5	0.5	0.05	30
S10	8	80	0.5	0.5	0.05	30

**Figure 3:**
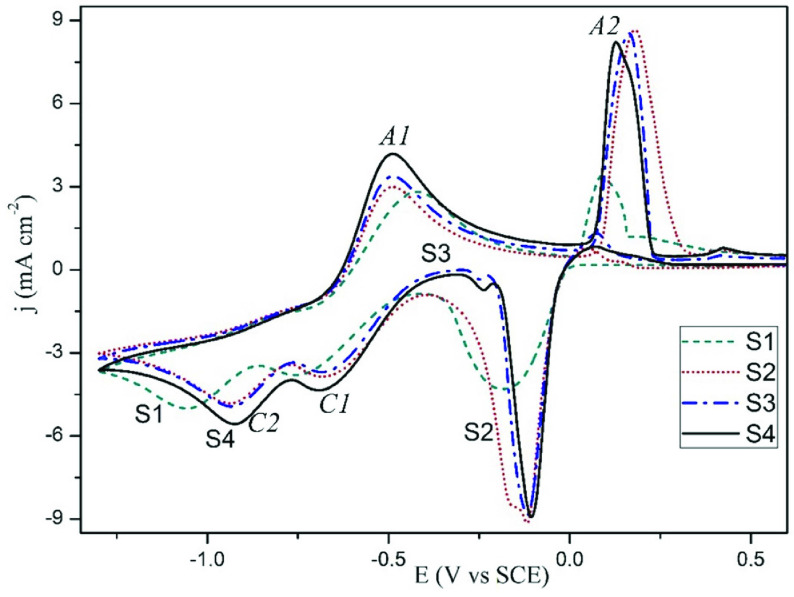
CV curves recorded in: (S1) KAg(CN)_2_ , (S2) KAg(CN)_2_+potassium pyrophosphate, (S3) KAg(CN)_2_+0.5 g L^-1^ KCN+potassium pyrophosphate, (S4-pyro electrolyte) KAg(CN)_2_+0.5 g L^-1^ KCN+potassium pyrophosphate+borax.

Figure 3 shows the CV curves of silver reduction from different electrolytes. KAg(CN)_2_ electrolyte (line S1 represents electrolyte S1) shows 2 irreversible cathodic reduction peaks at low and high cathodic potentials. The change of electrochemical reaction mechanism of electrodeposition of silver according to the cyanide concentration has been investigated in the literature. The dominant ion is *Ag(CN)^-^_2_* in the free cyanide concentration range of [CN^-^] ≤ 0.01 M [38]. That is accepted in this study as the low cyanide concentration limit. It should be highlighted here that Baltrûnas [39] showed that the active surface area of the cathode is dependent on the cyanide concentration. When [CN^-^] ≥ 0.5 M, almost 20% of the surface is active, whereas [CN^-^] ≤ 0.01 M, almost the entire surface area of the silver cathode is active. Considering this fact, low cyanide concentration electrolyte is allowed in the range of [CN^-^] ≤ 0.01 M (0.65 g.L^-1^) . In this range, at low cathodic potentials, the reduction occurs by charge transfer reaction subsequent to adsorption of cyano complex of Ag(I) as in the following Eq. (1a and 1b) [18]. At high cathodic potential, the direct reduction of Ag (I) cyano complex to metallic silver occurs, according to the Eq. (2) [18].

(1a)Ag(CN)2-⟷Ag(CN)2ads*-(adsorption)

(1b)Ag(CN)2ads*-+e-↔Ag+2CN-(charge transfer reaction)

(2)Ag(CN)2-+e-↔Ag+2CN-(direct reduction)

The addition of pyrophosphate (line S2) to this electrolyte (expressed as S1) depolarizes the reduction from –0.75 and –1.06 V to –0.68 ( *C1* ) and –0.94 V ( *C2* ), respectively. The same reduction behaviour of S2 is observed with the electrolyte containing 0.5 g L^-1^ KCN to this electrolyte, expressed as S3. It was observed that 0.5 g L^-1^ KCN does not influence the reduction potential associated with Baltrûnas’ results [38,39]. In anodic turn, the first anodic peak ( *A1* ) recorded at –0.48V indicates the formation of passive AgCN film and the area of this peak is increased very slightly with the addition of 0.5 g L^-1^ KCN. Nineva [40] also mentioned this peak. At the second anodic peak ( *A2* ) at ca. 0.2 V, silver oxide types are formed. When the cycle returns in the cathodic direction, a cathodic peak is recorded at –0.1 V before reaching the silver reduction peaks. This peak is the peak of the reduction of the silver oxide species formed in the anodic direction. A similar behaviour showing this peak was recorded in Krastev’s article [12] in which he studied silver electrodeposition from the ferrocyanide-thiocyanate electrolyte by cyclic voltammetry technique. Sodium tetraborate decahydrate (Borax) (line S4-borax added S3) increases the c.d. slightly without changing the reduction potentials as compared to the electrolyte without borax (S3).

**Figure 4:**
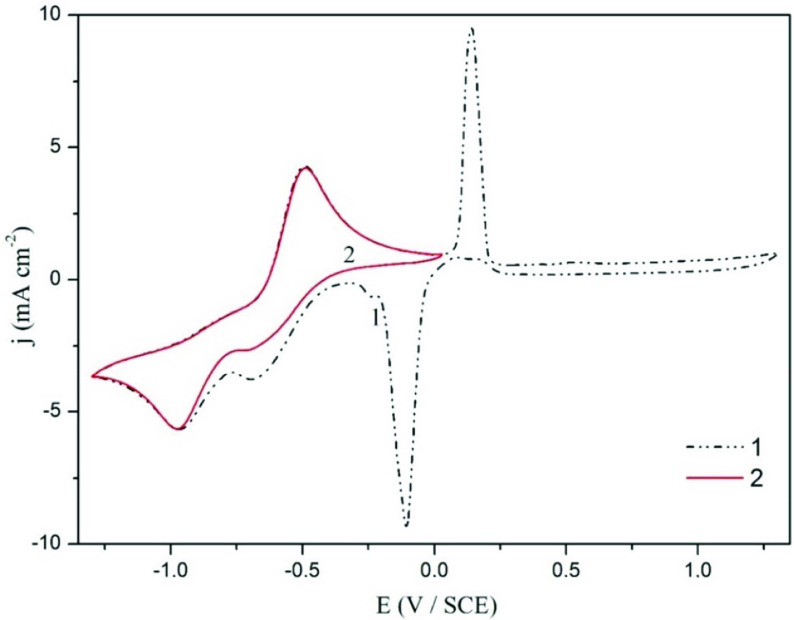
Voltamograms of S3 electrolyte (KAg(CN)_2_+0.5 g L^-1^ KCN+potassium pyrophosphate) recorded at different vertex potentials during the positive scan; Curve 1 terminated at 1.3 V, curve 2 terminated at 0.03 V [ν = 50 mV/s].

In Figure 4, cathodic peak recorded at –0.1 V was investigated again by changing the vertex voltage during the positive scan. Curve 1 shows the voltamogram of S3 electrolyte (KAg(CN)_2_ , pyrophosphate, and 0.5 g L^-1^ KCN) at the switching potential at 1.3 V during the positive scan. The oxide types of silver form approximately at 0.2 V in anodic direction. Distinctly in curve 2, since the switching potential is 0.03 V during the positive scan, the oxide type does not form. Therefore, the reduction peak of these oxide species recorded at –0.1 V in curve 1 is not observed in curve 2. This comparison shows that the reduction peak in the cathodic direction at –0.1 V is the reduction of the oxide type formed in the anodic direction.

**Figure 5:**
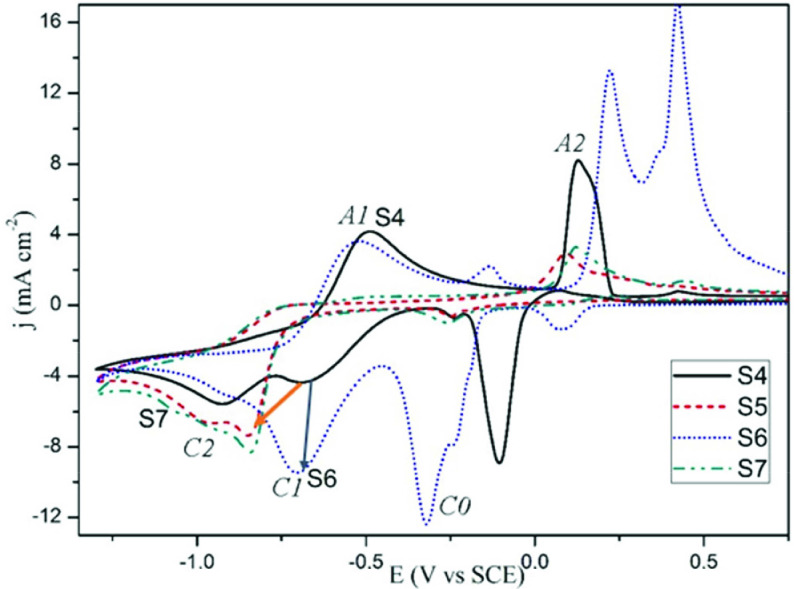
The effects of additives registered in CV curves: (S4) pyro electrolyte without additives, (S5) MBT+S4, (S6) PAT+S4, (S7) MBT+PAT+S4.

Figure 5 indicates the effects of additives on the reduction of silver cyano complex from the pyro electrolyte. The addition of MBT polarizes the reduction potentials to more cathodic potentials (line S5). Two cathodic peaks are recorded at –0.85 V and –0.97 V. This is the result of the passivation of the surface by the adsorption of the MBT additive to the active sides on the cathode surface. In the electrolyte S4, the formation of passive AgCN film recorded at –0.48 V is not observed in the presence of MBT. The formation of oxide species in the anodic zone also decreases. In other words, MBT makes the surface very passive. Therefore, the peak of the reduction of the anodic oxide species recorded in the cathodic direction (approximately at –0.25 V) is also relatively small. In microstructure investigations, the polarization effect of MBT is seen in the decrease in nodular growth. This is the result of the adsorption of MBT to the protrusions which tend to grow perpendicular to the cathode.

The electrolyte S6, containing PAT, results in a rapid increase in the c.d. of the cathodic reduction peak (–0.68 V), especially in the low cathodic potential region as compared with S4 electrolyte (no additive). This rapid increase is associated with microstructure results. The rapid c.d. increasing in narrow potential range means an increase in nucleation and a decrease in grain size as seen in the microstructure. Supporting this result, Saitou [11] investigated the effect of antimony on silver electrodeposition and showed that the rapid c.d. increase in the narrow potential range in cyclic voltammetry is positively related to the bright appearance. Additionally, the experimental results [11] showed that the effect of PAT is different than that of the levelling additives that prevent vertical growth as adsorbed in the cathode. In this study, microstructure investigations show that, on the addition of PAT in pyrophosphate-cyanide baths, the more dominant effect is that of grain refinement than blocking of vertical nodular growth. Another remarkable point in Figure 5 is that the addition of PAT (S6 curve) increases the c.d. values of the anodic oxide formation and silver dissolution by shifting the peaks to the more anodic potentials. This result is also related to the high grain refinement effect of PAT. Therefore, the reduction of peaks of this oxide type in the cathodic direction is polarized to ca. –0.3 V, and the peak areas increase.

It is seen that the addition of the MBT+PAT brightener couple indicated by line S7 forms a voltammogram similar to that of the only MBT containing electrolyte indicated by line S5. In other words, MBT’s polarization effect of adsorption is dominant, and the reduction peaks is recorded at –0.85 V and –1.05 V similarly as line S5. Also, simultaneously with the effect of MBT, the addition of PAT increases the c.d. values of polarized reduction.

**Figure 6:**
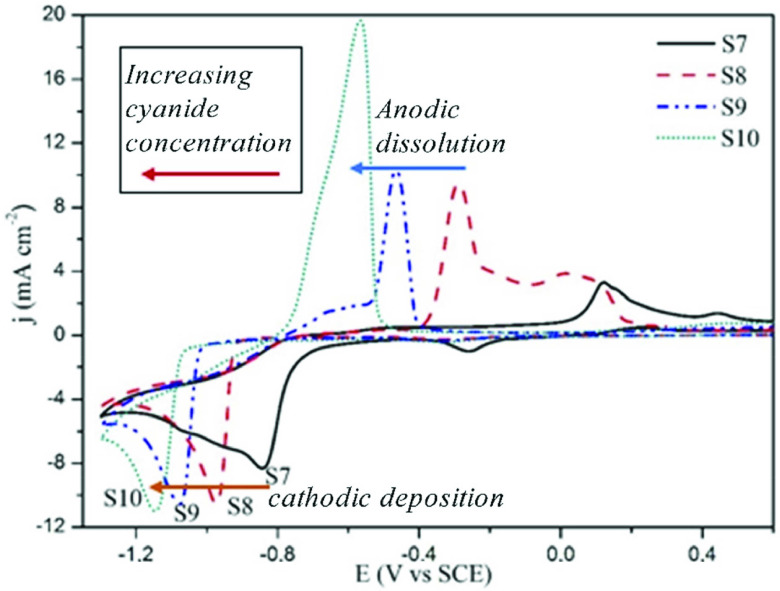
Cyanide concentration effect to silver reduction potential recorded in CV curves: (S7) pyro electrolyte+MBT+PAT, (S8) 5 g L^-1^ KCN+S7, (S9) 18 g L^-1^ KCN+S7, (S10) 30 g L^-1^ KCN+S7.

The influence of increasing the cyanide concentration on the MBT+PAT added pyro electrolyte behaviour is shown in Figure 6. In electrolytes containing the concentration of free cyanide above 5 g L^-1^ , the anodic oxide species, such as formed in the S7 electrolyte (MBT+PAT+pyro electrolyte), are not formed. Because of the increased concentration of free cyanide, silver dissolution occurs with a single anodic peak before the formation of silver oxide. These dissolution peaks in S8, S9, S10 lines recorded at –0.3, –0.47, –0.6 V, respectively. As the concentration of free cyanide increases, a single reduction peak occurs in the cathodic direction, and increased cyanide increases the polarization. The cathodic maximums of the electrolytes S7, S8, S9, S10 containing 0.5, 5, 18, 30 g L^-1^ KCN, are polarized to –0.85 V, –0.97 V, –1.08 V, and –1.15 V, respectively. The investigation on the mechanism of silver electrochemical reduction dependent on cyanide concentration between 0.01–1 M KCN confirmed that the dominated complex ion is *Ag(CN)* ^2-^_3_ . In these circumstances, the direct reduction as seen in Eq. (2) occurs after fast chemical reduction reaction [41]: *Ag(CN)* ^2-^_3_ +e^-^ ↔ Ag(CN)^-^_2_+ CN^-^.

It was stated that the polarization increase could be used as a criterion for the selection of an effective levelling agent, but polarization increase is not related only to good levelling properties [33]. Though MBT has a polarization effect, it does not provide a sufficient levelling effect on pyro electrolytes. In the FESEM study, it is also seen that the nodular growth is reduced but not prevented. It was observed here that, the sufficient levelling effect for smooth nano-sized microstructure in silver electrolytes could only be achieved with high cyanide electrolytes.

### 3.4. Chronoamperometry

**Figure 7:**
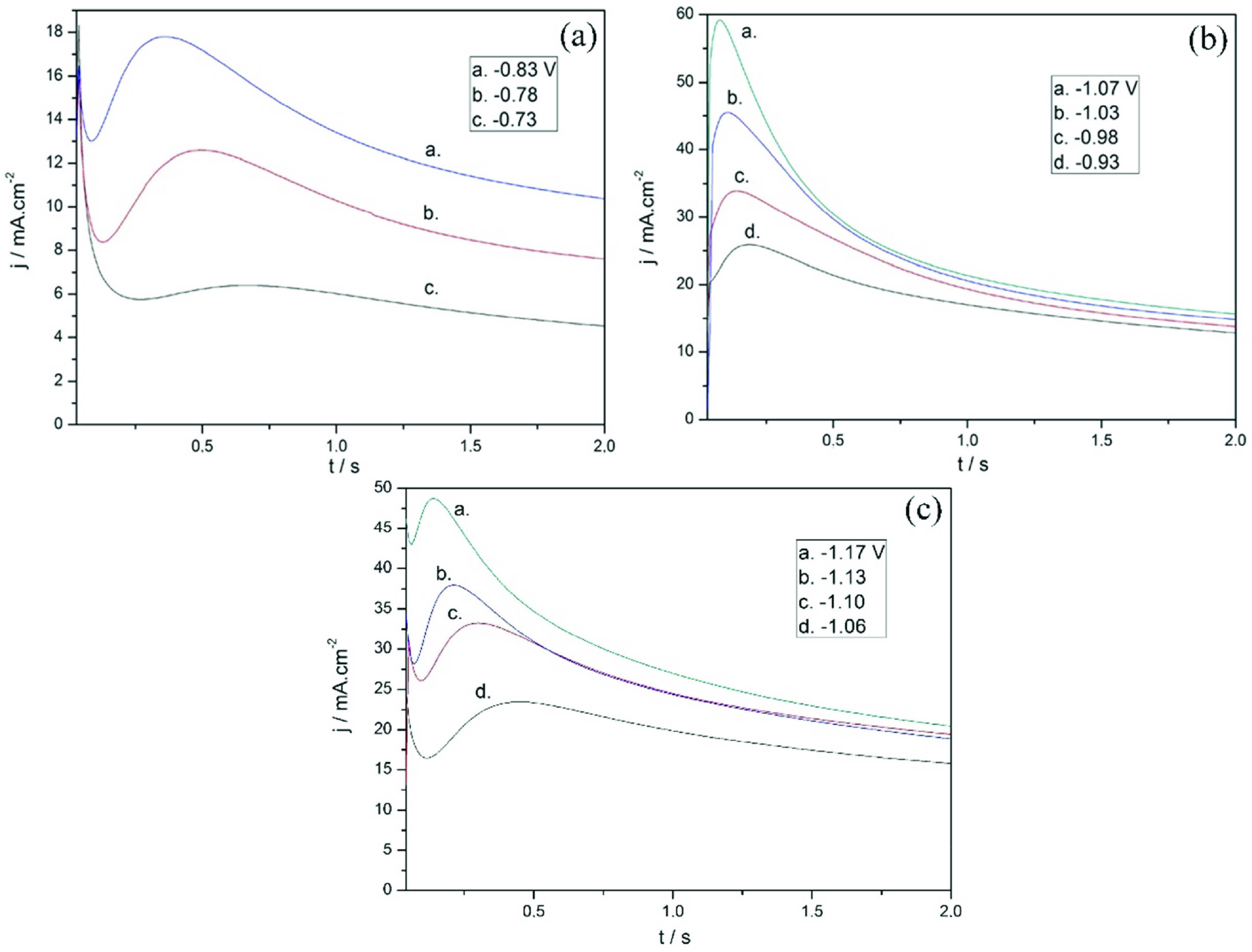
Current transients for silver electrodeposition with MBT+PAT from a) pyro electrolyte at low cathodic potentials, b) pyro electrolyte at high cathodic potentials, c) h-cya electrolyte.

Nucleation and growth mechanisms of silver electrodeposition were studied for additives containing pyro and hcya electrolytes. Figure 7 shows j-t curves indicating silver electrodeposition from the pyro electrolyte containing MBT + PAT for 2 cathodic peaks. Curves consist of 3 stages in the low cathodic region (Figure 7a). In the first stage, the decrease of the cathodic c.d. depends on the double-layer formation. In the second stage, the c.d. increases and reaches its maximum, indicating typical nucleation and growth. In the third stage, the c.d. decreases, and this stage is typical for the diffusion-controlled process. Then, the coating growth continues with a constant current. In the high cathodic potential region (Figure 7b), it is seen that the j-t curves have 2 stages without double-layer formation, also seen in Figure 7a. Figure 7c shows j-t curves in 3 stages for the silver electrodeposition from the high cyanide electrolyte containing MBT + PAT in a single cathodic region.

The transients fit the 3-dimensional (3D) classical nucleation and diffusional growth model of Scharifker and Hills. This model defines 2 different nucleation processes. “Instantaneous” nucleation is defined as nucleation that occurs in all active sites as soon as the potential is applied. “Progressive” nucleation refers to the formation of nuclei on active sites formed during electrodeposition. However, these definitions may confuse because, when the potential is applied, the nuclei never occur at once, they always form progressive [42]. Therefore, it would be appropriate to define instantaneous and progressive nucleation mechanisms as fast and slow nucleation, respectively [42].

In order to use the Scharifker theory, the current-time curves shown in Figure 7 were plotted for the first few seconds of coating formation. I^2^/I^2^_m_ vs. t/t_m_ graphs were derived from the experimental data in Figure 7. In Figures 7a and 7c, the first part of the double-layer formation was removed and the nucleation and growth mechanism was examined. I_m_ and t_m_ are the current and time corresponding to the maximum point of the peak in the graph. The theoretical curves for progressive and instantaneous nucleation were calculated by Eq. (3) and (4), respectively, which were derived by Scharifker [43]:

(3)I2Im2=1.2254ttm{1-exp(-2.3367(ttm)2)}2

(4)I2Im2=1.9542ttm{1-exp(-1.2564(ttm))}2

**Figure 8:**
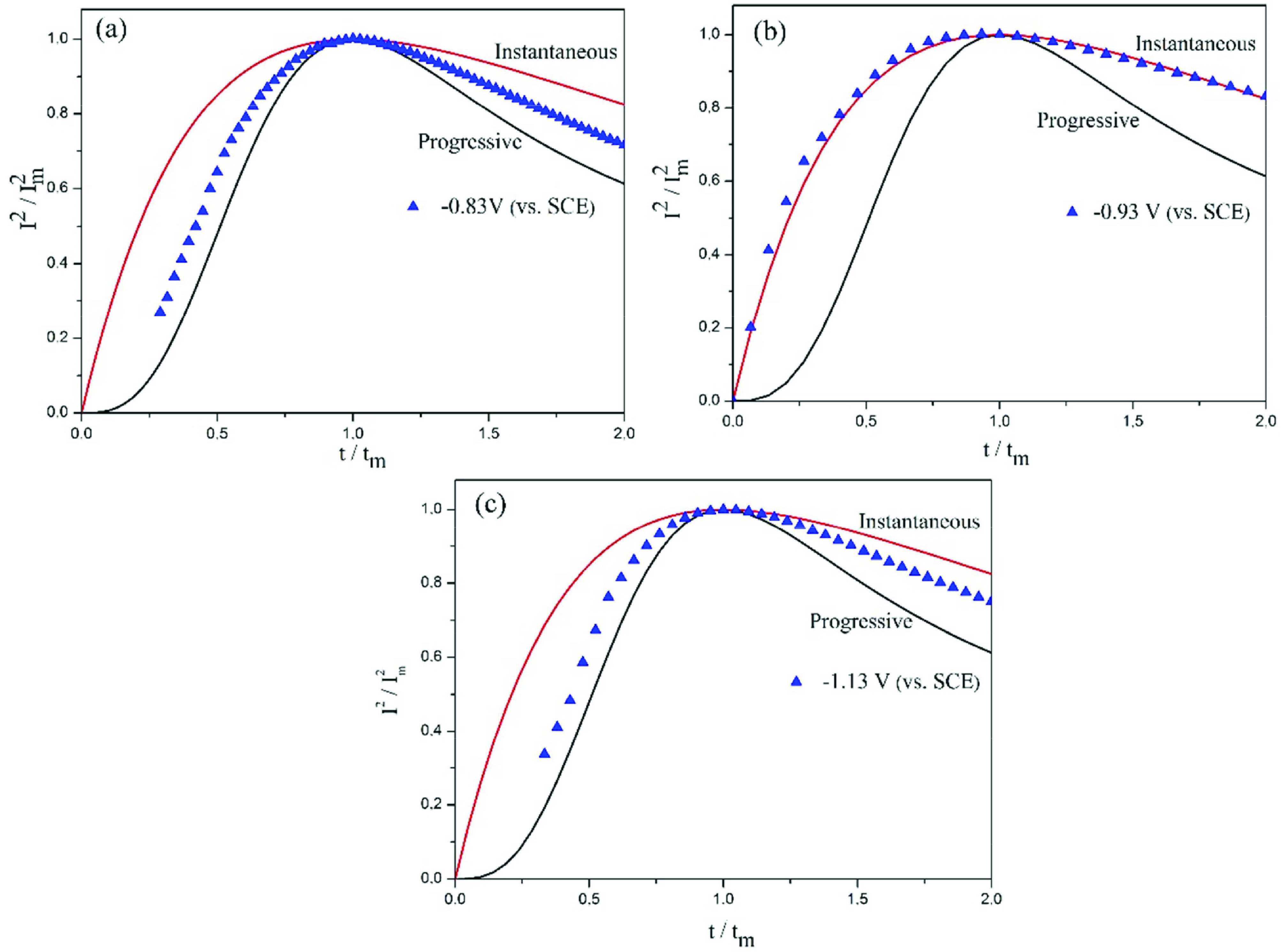
Comparison of the I^2^/I^2^_m_ vs. t/t_m_ experimental curves with the theoretical models for 3D nucleation and growth process. The experimental curves for silver electrodeposition with MBT+PAT from a) pyro electrolyte at low cathodic potentials, b) pyro electrolyte at high cathodic potentials, c) h-cya electrolyte are derived from current transients. The continuous lines correspond to the theoretically instantaneous (upper curve) and progressive (lower curve) nucleation and growth mechanism.

Considering the dimensionless I^2^/I^2^_m_ vs. t/t_m_ curves, the silver deposition from MBT+PAT including pyro electrolyte in low cathodic potentials in Figure 8a is formed by 3D progressive nucleation and growth mechanism. When high cathodic potentials are reached, the growth mechanism changes to 3D instantaneous nucleation and the growth mechanism is as seen in Figure 8b. On the other hand, the silver deposition from high concentration cyanide electrolyte that occurs only in high cathodic potentials fits 3D progressive nucleation and growth mechanism as shown in Figure 8c. It is seen that the curves do not fully fit the mechanism in the decreasing region of the current. This can be ascribed to the presence of some additional processes to 3D diffusion-controlled nucleation. Besides, j-t transition can be changed in the region of the decline of current to the shape of the growth centre (right circular cones, hemispheroids or paraboloids), while it is seen that the the geometry of the growth centre is independent of the increasing current portion of the transition [41].

The diffusion coefficient (D) of silver can be calculated from the nondimensional plots using the parameters i_m_ and t_m_ obtained from chronoamperometric curves. These parameters for instantaneous and progressive nucleation can be estimated by equation (5) and equation (6) [8, 9, 43], respectively:

(5)im2tm=0.1629D(nFC)2

(6)im2tm=0.2598D(nFC)2

For the silver electrodeposition reaction; n = 1, F is the Faraday constant, C is the concentration of silver species in the bath, D is the diffusion coefficient.

The diffusion coefficients of pyro electrolytes containing additive calculated using I-t measurements and Eq. (5) and (6) are, at low cathodic potentials 9.75 ×10^-6^ cm^2^s^-1^, and at high cathodic potentials 1.46 ×10^-5^ cm^2^ s^-1^. For h-cya electrolyte diffusion coefficient is 3.43 ×10^-5^cm^2^s^-1^ .

### 3.5. Reflectance analysis of the deposits

**Figure 9:**
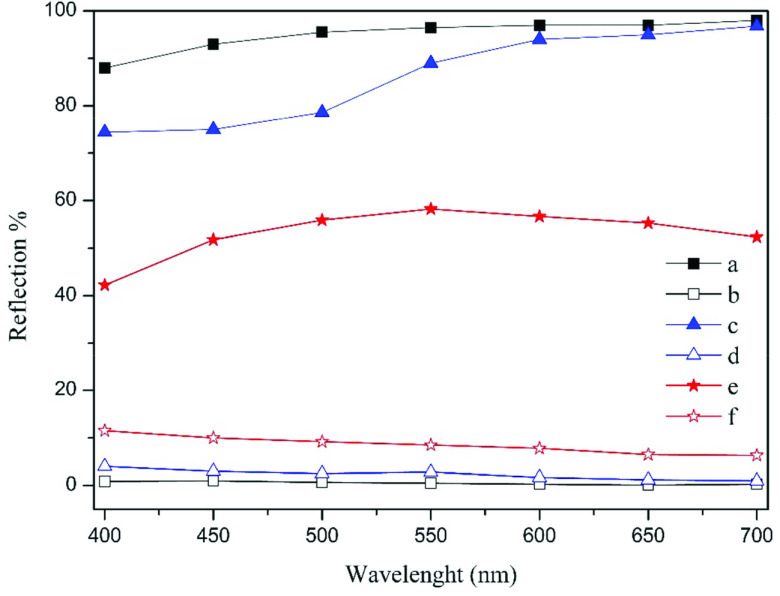
Specular reflection values of a) reference silver mirror, c) MBT+PAT added h-cya electrolyte coating, e) MBT+PAT added pyro electrolyte coating, and diffuse reflection values of b) reference silver mirror, d) MBT+PAT added h-cya electrolyte coating, f) MBT+PAT added pyro electrolyte coating.

Specular and diffuse reflection values of the reference silver and silver-coated samples were compared in Figure 9. As can be seen, the reference silver mirror shows a specular reflection close to 100%, and the proportion of diffuse reflection is very low. The sample obtained with h-cya electrolyte containing with MBT + PAT has a substantially specular reflectance above 550 nm and shows a reflection value very close to that of the reference silver mirror. The diffuse reflection value of this sample is also very low. In other words, the coating obtained with high cyanide concentration electrolyte containing MBT+PAT is very close in its reflectance to that of the reference mirror.

The sample obtained by electrodeposition in pyro electrolyte with MBT + PAT brighteners, as mentioned in the previous sections, is the brightest sample produced with low cyanide concentration electrolyte. It is semibright. In other words, it has a slightly hazy appearance. Therefore, the specular reflection value is much lower than the ideal reflectance of silver. The diffuse reflection value is higher as compared to the diffuse reflection value of the reference mirror. The specular reflection of this semigloss sample is around 50%–55%.

## 4. Conclusion

This research has compared and presented the effect of organic and inorganic brighteners used in cyanideand pyrophosphate-based silver electrolytes. It is demonstrated that the combination of PAT and MBT is an effective brightener couple for silver electrodeposition and KSeCN has less brightening effect on the above mentioned electrolytes. PAT has grain refinement effect, while MBT deactivates fast growing areas on the surface of the substrate by adsorbing and allowing the growth to progress in the recess areas, thereby reducing nodular growth which is also observed as polarization in cyclic voltammetry. On the other hand, the high concentration of cyanide polarizes the electrodeposition greatly. This strong polarizing effect provides sufficient leveling effect for forming mirror-bright coating. However, in pyrophosphate electrolytes, where free cyanide concentration was less than 0.01 M, brighteners did not cause sufficient leveling effect. Thus, these electrolytes produce semi-bright coatings.

At nucleation and growth level, the low cathodic potential electrodeposition of silver occurs in pyrophosphate electrolyte, which is found to proceed by 3D instantaneous nucleation. In high concentration cyanide electrolyte, this low cathodic potential electrodeposition polarized to high cathodic potentials and is not observed in low potential range. This high cathodic potential electrodeposition grows into with 3D progressive nucleation and diffusion-controlled growth mechanism.

To summarize the silver electrolytes investigated in this research for different applications, pyrophosphatecyanide bath contains 80 g L^-1^ KAg (CN)2+ 80 g L^-1^ pyrophosphate + 0.5 g L^-1^ KCN + 30 g L^-1^ borax + 0.05 g L^-1^ MBT, 0.5 g L^-1^ PAT components. It can be operated at 25 °C in the 1.0–6.0 A dm^-2^ c.d. range for the applications to obtain semi-bright finish while avoiding excessive use of cyanide. High concentration cyanide bath can be used for getting a mirror-bright finish in the 0.5–6.0 A dm^-2^ c.d. range at 25 °C. This bath comprises of 80 g L^-1^ KAg(CN)_2_+ 60 g L^-1^ KCN + 7.5 g L^-1^ KOH + 0.05 g L^-1^ MBT, 0.5 g L^-1^ PAT components.
